# Early urinary tract infection after kidney transplantation: timing, stent association, extended-spectrum β-lactamase infections, and clinical outcomes in a large cohort

**DOI:** 10.3389/frtra.2026.1902904

**Published:** 2026-08-11

**Authors:** Rehab Salah Fathy Nasr, Dieter Clemens Broering, Dalia Abdullah Ghalib Obeid, Reem Almaghrabi, Enas Mamdouh Mohamed Ali Hussein, Amir Nasser Teljabini, Manar Mohammed Saber Almadi, Tariq Zulfiqar Ali

**Affiliations:** 1 Kidney and Pancreas Health Centre, King Faisal Specialist Hospital and Research Centre, Riyadh, Saudi Arabia; 2 Department of Internal Medicine and Nephrology, Faculty of Medicine, Benha University, Benha, Egypt; 3 Organ Transplant Centre of Excellence, King Faisal Specialist Hospital and Research Centre, Riyadh, Saudi Arabia; 4 Transplant Research and Innovation, Organ Transplant Centre of Excellence, King Faisal Specialist Hospital and Research Centre, Riyadh, Saudi Arabia; 5 Transplant Infectious Diseases Section, King Faisal Specialist Hospital and Research Centre, Riyadh, Saudi Arabia; 6 Organ Transplant Service, King Faisal Specialist Hospital and Research Centre, Riyadh, Saudi Arabia

**Keywords:** extended-spectrum β-lactamase (ESBL), graft rejection, graft survival, kidney transplantation, ureteral stent, urinary tract infection

## Abstract

**Background:**

Urinary tract infection (UTI) including extended-spectrum β-lactamase (ESBL)-producing organisms is a common early complication after kidney transplantation. Increasing prevalence of ESBL may adversely affect graft and patient outcomes. This study aimed to evaluate the incidence, timing, predictors, and outcomes of UTI, including ESBL infection, after kidney transplantation.

**Materials and methods:**

We retrospectively studied 1,804 adult kidney transplant recipients (2016–2024) at a tertiary center. UTIs were analyzed for incidence, timing, microbiology, and predictors using multivariable logistic regression. Outcomes included rejection, graft loss, and mortality. ESBL and non-ESBL UTIs were compared, and long-term outcomes assessed using Cox regression.

**Results:**

UTI occurred in 693 recipients (38.4%) within the first year post-transplant with 57.4% occurring within 30 days and 84.2% within 90 days. UTIs were more common during ureteric stent indwelling than after removal (64.3% vs. 35.7%). Independent predictors included female sex, older age, deceased donor transplantation, pre-transplant UTI, and post-transplant urological complications. ESBL UTI were identified in 47.9% of patients and were associated with earlier infection, higher recurrence rates and occurred more frequently during stent indwelling (69.0% vs. 60.1%, *p* = 0.015). UTI was associated with higher rejection, graft loss, and mortality (*p* ≤ 0.005). ESBL UTI further increased rejection and graft loss. In adjusted analyses, ESBL UTI independently associated with death-censored graft loss (aHR 2.10, *p* = 0.047) and mortality (aHR 2.17, *p* = 0.025).

**Conclusions:**

UTIs are common early after kidney transplantation, particularly during ureteric stent indwelling. ESBL infections are independently associated with adverse graft outcomes and mortality, supporting targeted surveillance, antimicrobial stewardship, and ureteric stent management optimization.

## Introduction

Urinary tract infection (UTI) is among the most frequent complications after kidney transplantation, particularly within the first year after transplantation when immunosuppression is at its peak and urinary tract instrumentation is routinely performed. The reported incidence rates of UTIs ranges from 20% to >40% (1, 2). The use of ureteric stents, though beneficial in reducing urological complications, is a known risk factor for early and recurrent UTIs (3, 4).

In recent years, extended-spectrum β-lactamase–producing Enterobacteriaceae (ESBL-PE) have emerged as important uropathogens among kidney transplant recipients. These multidrug-resistant organisms present significant therapeutic challenges and are associated with recurrent infections, increased hospitalizations, and adverse graft outcomes. A meta-analysis reported that approximately 10% of kidney transplant recipients develop ESBL-PE–related UTIs, with higher prevalence observed in Asia (33%) and South America (17%) (5). Furthermore, several studies have reported that ESBL-producing strains account for approximately 40% of Escherichia coli UTIs in transplant patients, with particularly elevated rates in Asia (45%) and Europe (40%) (5–7).

ESBL-PE infections in kidney transplant recipients have been associated with a nearly threefold increased risk of UTI recurrence and relapse, as well as a higher incidence of biopsy-proven rejection (12.9% vs. 0.8%) (8). However, other studies have not demonstrated significant differences in acute rejection rates between ESBL-PE and non–ESBL-PE UTIs (9).

While some studies report increased risks of graft loss and mortality, the overall evidence remains inconsistent. Certain cohorts show increased morbidity, whereas others report comparable or even favorable long-term graft outcomes in ESBL-infected patients. These discrepancies likely reflect methodological limitations, including small sample sizes, single-center designs, and heterogeneous follow-up durations (1, 5, 7–12).

To date, large-scale evidence remains limited, with most studies on ESBL-PE UTIs in kidney transplant recipients confined to small single-center cohorts. The period during ureteric stent placement and following its removal—despite being recognized as a time of increased infection risk—remains underexplored. Furthermore, data on the impact of ESBL colonization on recurrence, rejection, graft loss, and mortality remain inconsistent.

To address these gaps, we analyzed 1,804 kidney transplant recipients at a tertiary center over 8 years, evaluating UTI incidence, risk factors, and the impact of ESBL-positive infections on recurrence, rejection, and long-term patient and graft outcomes.

This large single-center study provides additional insights into the effect of early post-transplant infections on long-term outcomes and may inform targeted antibiotic stewardship and monitoring and interventional strategies.

## Materials and methods

This retrospective cohort study was conducted at King Faisal Specialist Hospital and Research Centre in accordance with the Declaration of Helsinki and approved by the institutional review board (IRB No. 2255740).

Adult kidney transplant recipients (≥18 years) transplanted between January 2016 and June 2024 were included. Patients with incomplete records, missing urine cultures, or follow-up <1 year were excluded. The final cohort comprised 1,804 recipients, categorized into UTI and non-UTI groups, with further stratification into ESBL-positive and ESBL-negative UTI subgroups for short- and long-term outcome analyses.

### Definitions of variables and outcomes

- Definition of symptomatic UTI: Positive urine culture with ≥10^5^ CFU/mL of uropathogen(s) in urine culture accompanied by clinical signs or symptoms (e.g., dysuria, frequency, urgency, suprapubic tenderness, fever, or leukocytosis) (13, 14).
- Recurrent UTI: Two or more UTI episodes within 6 months or ≥3 episodes within 1-year, with at least one episode confirmed by urine culture to establish an infectious etiology. The diagnosis of recurrent UTI requires that each episode be separated by a period of complete clinical resolution (15).
- ESBL status: Defined by the isolation of a gram-negative bacterium that demonstrates resistance to extended-spectrum β-lactam antibiotics (Penicillin, cephalosporins and Aztreonam) due to the production of ESBL enzymes (16).
- Graft loss: Defined as either return to dialysis or re-transplantation.

### Foley catheter and stent policy

All recipients underwent Foley catheter insertion during transplantation, typically removed between postoperative days 5–7. A double J stent was routinely inserted and removed approximately 6 weeks after transplantation.

### Infection prophylaxis

Patients received trimethoprim-sulfamethoxazole for Pneumocystis prophylaxis for 6 months post-transplant, along with cytomegalovirus prophylaxis.

### Urine culture policy

Urine cultures were routinely obtained during the first 6 months after transplantation at scheduled clinic visits. Both symptomatic UTIs and episodes of asymptomatic bacteriuria have been captured in the dataset. Beyond 6 months, cultures were performed only in patients with symptoms suggestive of UTI.

### Treatment of UTI

Symptomatic UTIs were consistently treated. Asymptomatic UTIs were treated only during the first 6 months post-transplant. Patients with recurrent UTIs underwent urological evaluation for structural or functional abnormalities.

### Immunosuppression protocol

Induction therapy consisted of ATG, basiliximab, or no induction based on immunological risk. Maintenance therapy generally included corticosteroids, mycophenolate mofetil, and tacrolimus.

### Diagnosis of rejection

Indication biopsies were performed for suspected rejection or unexplained graft dysfunction. Rejection episodes were graded according to the Banff classification (17).

### Outcomes

Primary outcomes included the prevalence, predictors, and microbiological profile of first-year UTIs, as well as associated graft function, rejection, and patient and graft survival.

Secondary outcomes included the prevalence, predictors, microbiology, and clinical outcomes of ESBL-producing UTIs compared with non-ESBL UTIs.

### Statistical analysis

Descriptive statistics summarized the cohort. Group comparisons used the Welch *t*-test, Pearson chi-squared test, or Fisher exact test, as appropriate. Multivariable logistic regression assessed factors associated with first-year UTI. The model included age, sex, donor type, ABO incompatibility, HLA mismatch, rituximab therapy, induction therapy, pre-transplant UTI, and post-transplant urological complications, as displayed in Table 2.

For death-censored graft loss and all-cause mortality, the primary Cox models treated first UTI as a time-updated exposure that changed from unexposed to exposed on the recorded first-UTI day. Models used time since transplantation as the time scale and included age (per 10 years), sex, donor type, and pre-transplant UTI. A time-updated sensitivity analysis separated first-episode non-ESBL and ESBL UTI. Because the date of a later ESBL episode was unavailable, the broader any-ESBL-during-year-1 variable was analyzed only in a day-365 landmark model.

First-UTI incidence was also summarized per 1,000 patient-days before and after stent removal, and stent status was entered as a time-varying covariate. Proportional-hazards assumptions were assessed using scaled Schoenfeld residuals. Donor type violated proportional hazards in the mortality and stent models and was therefore used as a stratification factor in those models. Variance-inflation factors assessed collinearity. Three UTI recipients with missing first-UTI timing were excluded from time-updated analyses; ten with missing first-episode ESBL status were censored at the infection date in subtype analyses. Statistical significance was set at *p* < 0.05. Analyses used R 4.5.2.

## Results

### Patients' demographical and clinical data by UTI status

This study included 1,804 adult patients who underwent kidney transplant between 2016 and 2024. Their mean age was 46 years, and 62.2% of them were men. Tacrolimus-based triple therapy was administered to 1,742 (96.56%). The average duration of ureteric stent placement was 41.77 ± 16.46 days.

Among the patients, 693 (38.4%) developed UTI at least once within the first year after transplantation. Patients who developed UTIs were older (47.6 vs. 44.9 years, *p* < .001), and less likely to be male (42.9% vs. 74.3%, *p* < 0.001). Diabetes and urological causes of end stage renal disease (ESRD) were more frequent among the UTI group (31.2% and 6.6%, respectively) (Table 1). Recipients with UTI were more likely to have undergone multiple transplants (≥2: 10.4% vs. 6.1%, *p* = 0.002), received deceased donor grafts (15.3% vs. 9.5%, *p* < 0.001), been ABO-incompatible (8.1% vs. 5.4%, *p* = 0.024), and have a positive T- and B-cell flow crossmatch (5.9% vs. 3.2%, *p* = 0.006). A higher degree of HLA mismatch (>4) was also associated with increased UTI risk (27.6% vs. 23.1%, *p* = 0.034).

**Table 1:** Patients’ clinical and demographical characteristics by UTI status within the first year after transplantation.

Characteristic	Overall	UTI at 1 year post transplant		
		No UTI detected	UTI detected	*p*-value^a^
	*N* = 1,804	*N* = 1,111	*N* = 693	
Age, mean ± SD	45.96 ± 15.79	44.94 ± 15.39	47.59 ± 16.27	<0.001
Sex, male (%)	1,122 (62.20)	825 (74.26)	297 (42.86)	<0.001
ESRD cause, *n* (%)				0.001
Diabetes	496 (27.49)	280 (25.20)	216 (31.17)	
Glomerulonephritis	338 (18.74)	215 (19.35)	123 (17.75)	
Unknown	769 (42.63)	507 (45.63)	262 (37.81)	
Urological	91 (5.04)	45 (4.05)	46 (6.64)	
Others	110 (6.10)	64 (5.76)	46 (6.64)	
Number of transplants, *n* (%)				0.002
1	1,664 (92.24)	1,043 (93.88)	621 (89.61)	
2	131 (7.26)	66 (5.94)	65 (9.38)	
3	8 (0.44)	2 (0.18)	6 (0.87)	
4	1 (0.06)	0 (0.00)	1 (0.14)	
LD/DD, *n* (%)				<0.001
LD	1,593 (88.30)	1,006 (90.55)	587 (84.70)	
DD	211 (11.70)	105 (9.45)	106 (15.30)	
Duration of stent, mean ± SD	41.77 ± 16.46	42.40 ± 11.94	40.76 ± 21.80	<0.001
ABO incompatible, *n* (%)	116 (6.43)	60 (5.40)	56 (8.08)	0.024
Cross match				
B+ ve, *n* (%)	123 (6.82)	69 (6.21)	52 (7.50)	0.286
T + B+ ve, *n* (%)	80 (4.44)	36 (3.24)	41 (5.92)	0.006
DSA+ ve, *n* (%)	377 (20.92)	218 (19.62)	158 (22.80)	0.106
HLA mismatch, *n* (%)				0.034
(0–4)	1,356 (75.17)	854 (76.87)	502 (72.44)	
(>4)	448 (24.83)	257 (23.13)	191 (27.56)	
Desensitization				
Rituximab, *n* (%)	265 (14.69)	148 (13.32)	117 (16.88)	0.038
IVIG, *n* (%)	247 (13.69)	132 (11.88)	115 (16.59)	0.005
TPE, *n* (%)	146 (8.09)	83 (7.47)	63 (9.09)	0.22
Induction therapy^b^				<0.001
ATG	1,339 (74.31)	770 (69.37)	569 (82.23)	
Basiliximab	335 (18.59)	244 (21.98)	91 (13.15)	
No induction	128 (7.10)	96 (8.65)	32 (4.62)	
Total ATG dose (mg), mean ± SD	183.34 ± 150.19	172.71 ± 153.43	200.39 ± 143.33	<0.001
Pre-transplant UTI, *n* (%)	237 (13.14)	111 (9.99)	126 (18.18)	<0.001
Urological complications				
Ureteric stricture, *n* (%)	44 (2.44)	8 (0.72)	36 (5.19)	<0.001
Ureteric leak, *n* (%)	26 (1.44)	4 (0.36)	22 (3.17)	<0.001
Surgical intervention for urological complications, *n* (%)	24 (1.33)	2 (0.18)	22 (3.17)	<0.001
Nephrostomy tube insertion *n* (%)	49 (2.72)	9 (0.81)	40 (5.77)	<0.001
Rejection, *n* (%)	258 (14.30)	138 (12.42)	120 (17.32)	0.005
Graft loss, *n* (%)	40 (2.22)	16 (1.44)	24 (3.46)	0.005
Creatinine (µmol/L) at 1 year	100.91 ± 59.39	100.03 ± 45.11	102.35 ± 77.19	<0.001
Died, *n* (%)	60 (3.33)	25 (2.25)	35 (5.05)	0.001

IVIG, intravenous immunoglobulin; TPE, therapeutic plasma exchange; ATG, antithymocyte globulin; DD, deceased donor; LD, living donor; DSA, donor specific antibody; ESRD, end stage renal disease.

a Welch two sample *T* test; Pearson's Chi-squared test; Fisher's exact test.

b 2 Patients received *CAMPATH*.

Desensitization with rituximab and intravenous immunoglobulin (IVIG) was more frequent among patients who developed UTI (*p* = 0.038 and *p* = 0.005, respectively). Induction with anti-thymocyte globulin (ATG) was more common in the UTI group (82.2% vs. 69.4%, *p* < 0.001), with higher total ATG doses observed in these patients (200.4 ± 143.3 vs. 172.7 ± 153.4 mg, *p* < 0.001).

UTI was more prevalent among those who had history of UTIs prior to kidney transplant (18.2% vs. 10.0%, *p* < 0.001).

Post-transplant urological complications were significantly more frequent in the UTI group, including ureteric stricture (5.2% vs. 0.7%, *p* < 0.001), ureteric leak (3.2% vs. 0.4%, *p* < 0.001) and required surgical intervention (3.2% vs. 0.2%, *p* < 0.001), and nephrostomy tube insertion (5.8% vs. 0.8%, *p* < 0.001).

**Figure 1:**
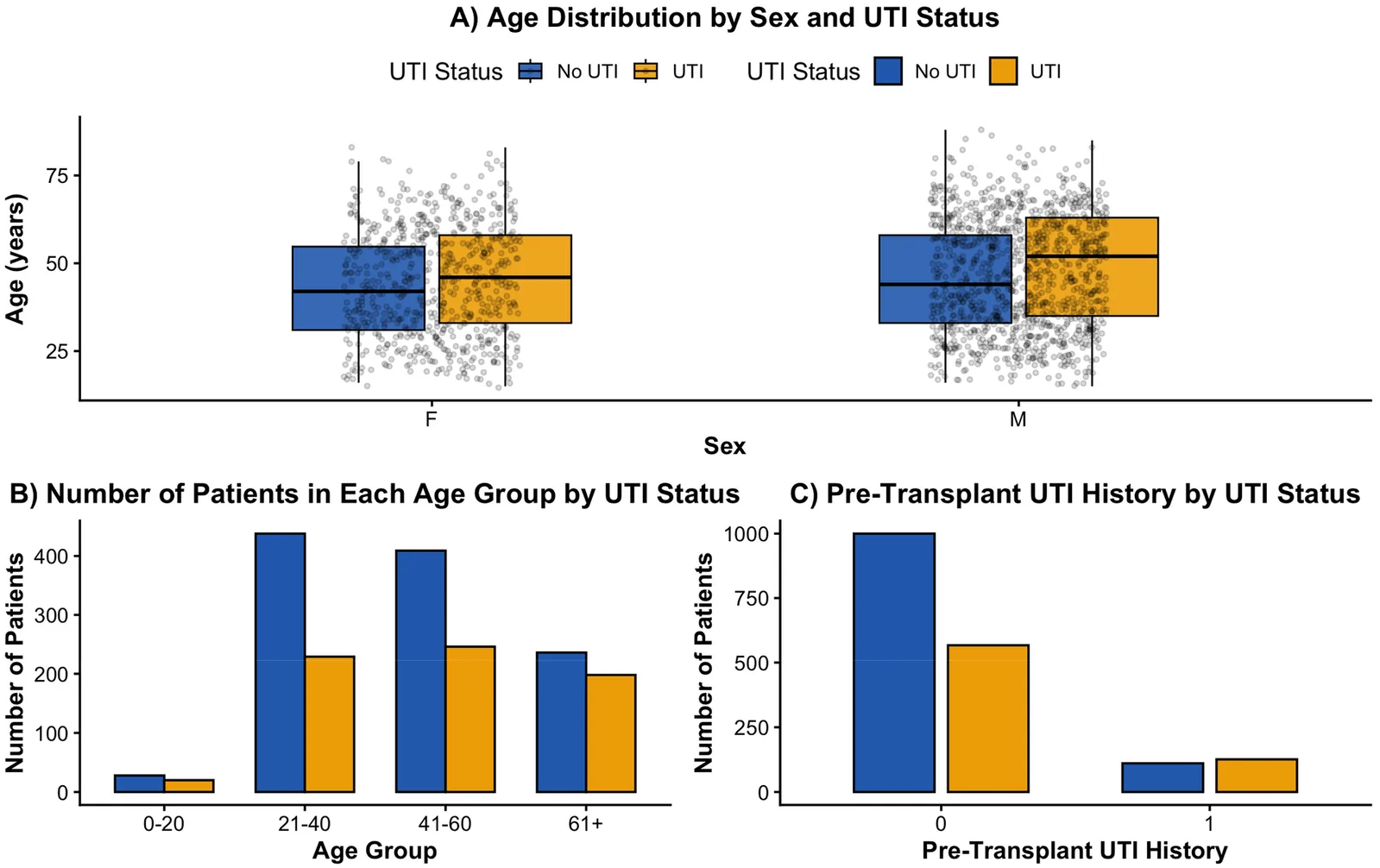
Distribution of clinical characteristics by UTI status within the first year following transplantation and stent removal. **(A)** Age distribution by sex and UTI status. **(B)** Distribution of age groups stratified by UTI status. **(C)** Frequency of UTI cases by history of pre-transplant UTI. Colors indicate UTI status (blue = no UTI; yellow = UTI). Boxplots represent medians and interquartile ranges; violin plots display the full distribution of the data.

At 1 year, UTI was associated with higher crude rates of rejection (17.3% vs. 12.4%, *p* = 0.005), graft loss (3.5% vs. 1.4%, *p* = 0.005), and mortality (5.1% vs. 2.3%, *p* = 0.001). Mean serum creatinine differed by only 2.3 micromol/L (102.35 vs. 100.03 micromol/L), was not statistically significant by the prespecified Welch *t*-test (*p* = 0.501), and is unlikely to be clinically meaningful. These characteristics are summarized in Table 1 and Figure 1.

### Timing of the first episode

A total of 396 patients (57.4%) developed a UTI within the first 30 days post-transplant. An additional 185 (26.8%) developed UTI between days 31 and 90, while 109 (15.7%) developed UTI after day 90; data were missing for three patients.

Among the 690 recipients with complete timing data, 444 (64.3%) had their first UTI while the ureteric stent was *in situ* and 246 (35.7%) after removal. These events occurred over 65,572 and 373,640 patient-days, respectively, corresponding to crude rates of 6.77 and 0.66 per 1,000 patient-days. Forty-two post-removal events occurred within 7 days (4.50/1,000 patient-days), compared with 204 later events (0.56/1,000 patient-days). After accounting for time since transplantation and stratifying by donor type, stent status was not associated with first UTI (aHR 0.98, 95% CI 0.68–1.42; *p* = 0.929). Three recipients lacked timing data.

### Factors associated with urinary tract infections in kidney transplant recipients

In multivariable analysis, female sex, older age, and deceased donor transplantation were associated with greater odds of first-year UTI (Table 2). Pre-transplant UTI was also associated with UTI, and post-transplant urological complications showed the largest adjusted association. ABO incompatibility, HLA mismatch, rituximab use, and induction regimen were not statistically significant in the adjusted model.

**Table 2:** Factors associated with first-year urinary tract infection (UTI) in univariable and multivariable logistic regression analyses.

Characteristic	Univariable OR^a^ (95% CI)^a^	*p* value	Multivariable OR^a^ (95% CI)^a^	*p* value
Age in years	1.01 (1.00–1.02)	<0.001	1.02 (1.01–1.02)	<0.001
Sex (male as ref)				
Female	3.85 (3.15–4.71)	<0.001	4.17 (3.35–5.20)	<0.001
Cause of ESRD				
Diabetes (Ref)				
Glomerulonephritis	0.74 (0.56–0.98)	0.039		
Unknown	0.67 (0.53–0.84)	<0.001		
Urological	1.33 (0.85–2.08)	0.22		
Others	0.93 (0.61–1.41)	0.74		
DD (vs. LD)^b^	1.73 (1.30–2.31)	<0.001	1.80 (1.25–2.58)	0.002
ABO incompatibility	1.54 (1.05–2.25)	<0.001	1.57 (0.96–2.57)	0.073
HLA mismatch (vs. >4)				
0–4 Mismatches	0.79 (0.64–0.98)	0.034	1.07 (0.81–1.41)	0.634
Rituximab therapy	1.32 (1.01–1.72)	0.038	0.74 (0.52–1.06)	0.073
Induction therapy				
ATG	Ref		Ref	
Basiliximab	0.50 (0.39–0.65)	<0.001	0.76 (0.56–1.03)	0.078
No induction	0.45 (0.29–0.68)	<0.001	0.67 (0.41–1.02)	0.067
Pre-transplant UTI	2.00 (1.52–2.64)	<0.001	1.97 (1.46–2.65)	<0.001
Urological complications (ureteric stricture &leak)	6.74 (3.49–14.4)	<0.001	8.43 (4.20–18.5)	<0.001

ESRD, end stage renal disease; DD, deceased donor; LD, living donor.

a OR, odds ratio; CI, confidence interval.

b Reference categories for categorical variables are listed after “vs.”

### Microbiological profile

Among 693 patients with UTI, 332 (47.9%) experienced at least one ESBL-positive episode, including 287 (41.4%) with ESBL detected in the first UTI episode. Blood cultures were positive in 7.3% of cases, and the median number of UTI episodes was 2 (IQR 1–5).

**Figure 2:**
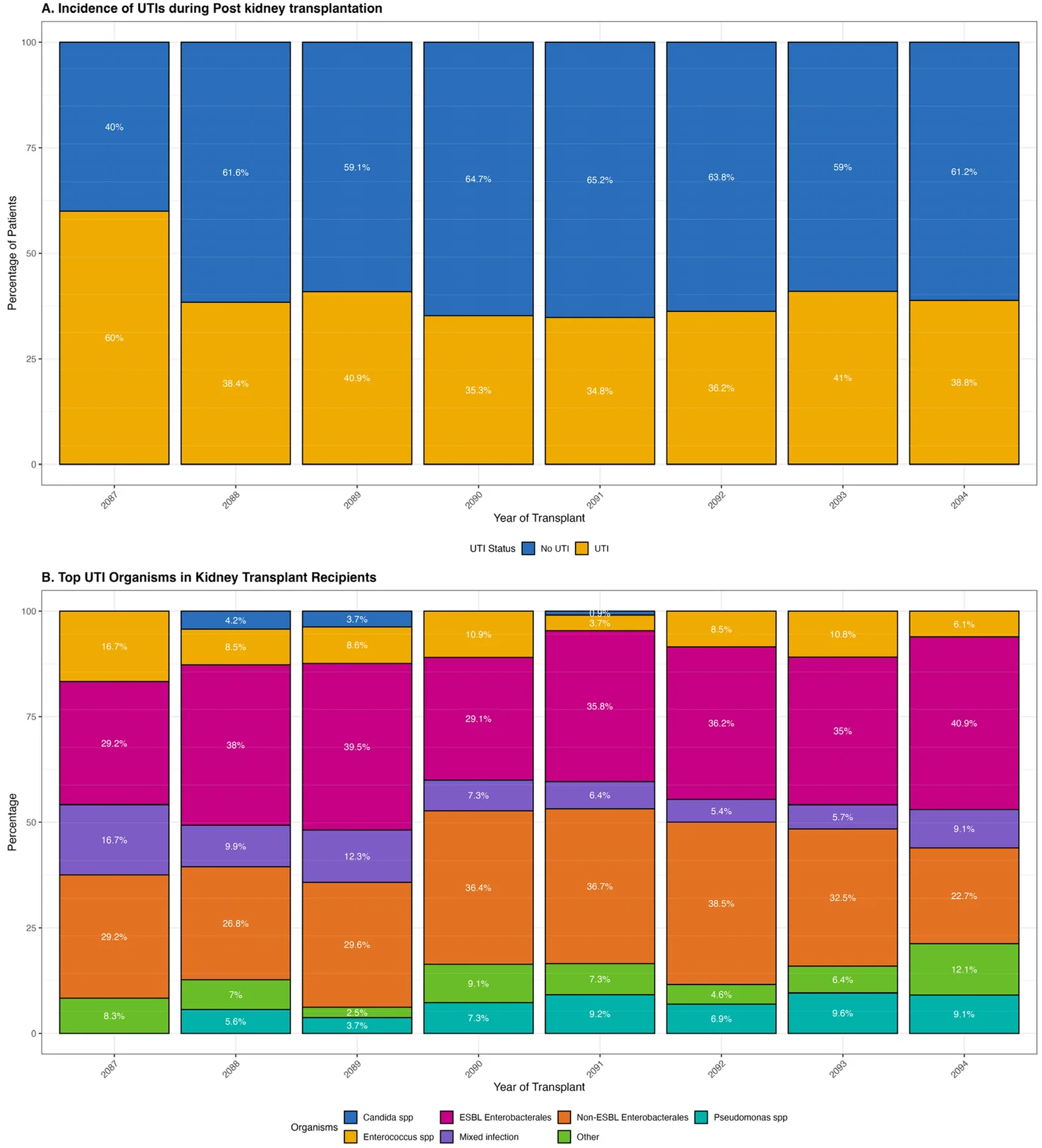
Trends in post stent (UTIs) and causative organisms in kidney transplant recipients. **(A)** Yearly prevalence of UTIs occurring within the first year after transplantation after ureteral stent removal among kidney transplant recipients from 2017 to 2024. **(B)** Distribution of top UTI-causing organisms among infected patients by year of transplant. UTI status (panel A) is color-coded as blue (No UTI) and yellow (UTI).

Gram-negative organisms predominated, with a high burden of ESBL-producing strains. Klebsiella spp. and Escherichia coli were the most common pathogens, followed by Enterococcus spp. and Pseudomonas spp. Further microbiological details are summarized in Table 3 and Figure 2.

**Table 3:** Microbiological profile.

Characteristic	No. (%) or median (IQR)
Total patients with UTI	693
Any ESBL-all 1st year	332 (47.91)
ESBL at first episode	287 (41.41)
Positive blood culture at first UTI	50 (7.25)
Number of UTI episodes within 1 year	2 (1–5)
Escherichia coli infection	
Non-ESBL	127 (18.33)
ESBL	152 (21.93)
Both	26 (3.75)
Klebsiella species infection	
Non-ESBL	151 (21.79)
ESBL	146 (21.07)
Both	52 (7.50)
Enterobacter species infection	
Non-ESBL	7 (1.01)
ESBL	24 (3.46)
Both	1 (0.14)
Others	
*Pseudomonas species*	95 (13.71)
*Acinetobacter species*	20 (2.89)
*Enterococcus species*	98 (14.14)
*Staphylococcus species*	18 (2.60)
*Streptococcus species*	22 (3.17)

### Clinical characteristics and outcomes according to ESBL status

ESBL-producing organisms were identified in 332 patients. Baseline characteristics were similar between the ESBL and non-ESBL groups, although female sex was more common in the ESBL group (60.8% vs. 53.9%, *p* = 0.054). Patients with ESBL UTIs experienced significantly more recurrent infections (*p* < 0.001), while ESRD etiology, transplant variables, and urological outcomes were comparable between groups (Table 4).

**Table 4:** Clinical characteristics and outcomes according to ESBL status among recipients with UTI.

Characteristic	Non ESBL (*N* = 361)	ESBL (*N* = 332)	*p* value
Demographic characteristics			
Age, year (IQR)	50.0 (34.0–61.5)	48.5 (34.0–60.5)	0.425
Female sex, no. (%)	194 (53.89)	202 (60.84)	0.054
No. of UTI episodes within 1 year	2.00 (1.00–4.00)	3.00 (2.00–6.00)	<0.001
Pretransplant UTI no. (%)	63 (17.5)	63 (19.0)	0.60
Positive blood culture no. (%)	16 (4.48)	34 (10.24)	0.01
Cause of ESRD no. (%)			0.46
Diabetes	117 (32.50)	99 (29.82)	
Glomerulonephritis	65 (18.06)	58 (17.47)	
Urological	23 (6.39)	23 (6.93)	
Unknown	134 (37.22)	128 (38.55)	
Others	21 (5.83)	24 (7.23)	
Transplant characteristics, no. (%)			
DD, no. (%)	56 (15.51)	50 (15.06)	0.916
HLA mismatch >4	99 (27.50)	91 (27.41)	0.401
ABO incompatible	31 (8.61)	25 (7.53)	0.702
Positive DSA	73 (20.28)	85 (25.60)	0.213
Induction therapy, no. (%)			0.758
ATG	292 (81.34)	276 (83.13)	
Basiliximab	51 (14.21)	40 (12.05)	
No induction	16 (4.46)	16 (4.82)	
Urological complication no. (%)			
Surgical intervention	10 (2.78)	12 (3.61)	0.676
Nephrostomy tube insertion	17 (4.72)	23 (6.93)	0.297
Ureteric stricture	15 (4.17)	21 (6.33)	0.272
Ureteric leak	8 (2.22)	14 (4.22)	0.218
UTI/Stent status			
Stent duration overall days (IQR)	41.0 (35.0–44.0)	41.0 (34.0–44.0)	0.686
UTI during stent presence no (%)	215 (60.06)	229 (68.98)	0.015
UTI after stent removal no (%)	143 (39.94)	103 (31.02)	
Unknown	3	0	
UTI during stent presence, days^a^ median (IQR)	14.00 (10.00–23.00)	14.00 (11.00–20.00)	0.517
UTI after stent removal, days^b^ median (IQR)	61.00 (14.00–171.00)	25.00 (9.00–61.00)	<0.001
Outcomes			
Acute rejection, no. (%)	54 (14.96)	66 (19.88)	0.089
ABMR, no. (%)	5 (1.39)	2 (0.60)	
TCMR, no. (%)	28 (7.78)	33 (9.94)	
Borderline, no. (%)	16 (4.44)	28 (8.43)	
Mixed (TCMR + ABMR), no. (%)	4 (1.11)	3 (0.90)	
Graft loss, no. (%)	11 (3.05)	13 (3.92)	0.542
Death, no. (%)	19 (5.28)	16 (4.82)	0.863
Creatinine µmol/L at 1 year (IQR)	87.00 (74.0–103.0)	88.0 (70.0–113.0)	0.272

ABMR, antibody-mediated rejection; TCMR, T-cell mediated rejection; DD, deceased donor; ATG, anti thymocyte globulin; DSA, donor specific antibody; ESRD, end stage renal disease. Fisher exact tests were used for rejection, graft loss, and death. One non-ESBL recipient with rejection lacked a classified rejection subtype.

a From date of transplant to UTI while stent was present.

b From date of stent removal to UTI.

Stent duration was similar overall (median 41 days, *p* = 0.686), but ESBL infections occurred more frequently during stent indwelling (69.0% vs. 60.1%, *p* = 0.015). Following stent removal, ESBL UTIs developed significantly earlier than non-ESBL UTIs (25 vs. 61 days, *p* < 0.001).

The ESBL group had numerically higher rates of biopsy-proven rejection (19.9% vs. 15.0%; Fisher *p* = 0.089) and graft loss (3.9% vs. 3.0%; Fisher *p* = 0.542), whereas mortality (4.8% vs. 5.3%; Fisher *p* = 0.863) and one-year serum creatinine were similar between groups.

### Overall long-term patient and graft outcomes

**Figure 3:**
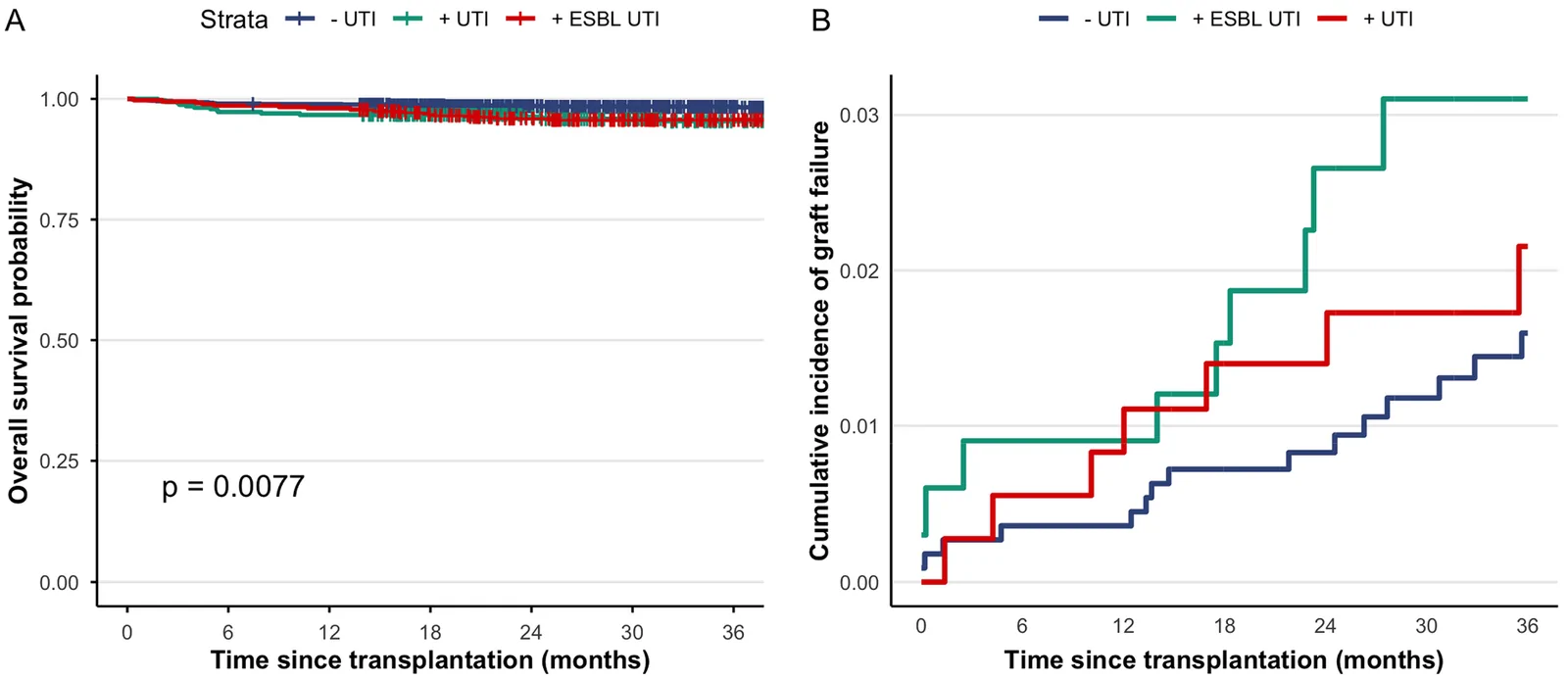
Three-year survival and graft failure outcomes according to UTI status after transplantation. **(A)** Kaplan–Meier estimates of overall survival probability since transplantation. **(B)** Cumulative incidence of graft failure accounting for the competing risk of death. Survival and incidence curves are shown for patients without UTI, with non-ESBL UTI, and with ESBL UTI during the first year after transplantation. Time is presented in months, truncated at 36 months of follow-up.

**Figure 4:**
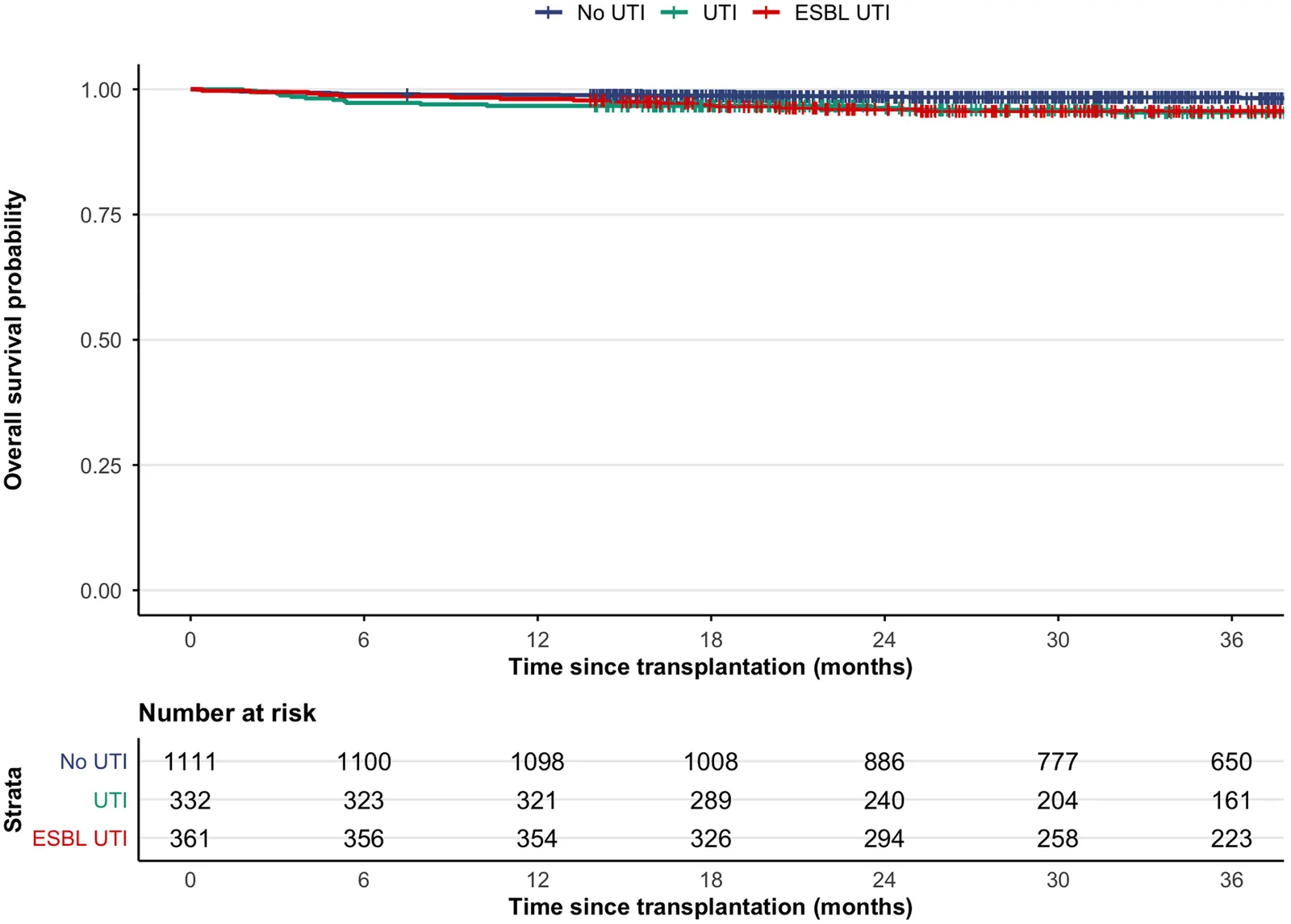
Three-year overall survival according to UTI status (with number at risk). This figure presents Kaplan–Meier estimates of overall survival up to 36 months following transplantation, stratified by UTI status during the first year after transplantation (no UTI, non-ESBL UTI, and ESBL UTI), along with the corresponding numbers at risk at predefined time points.

**Figure 5:**
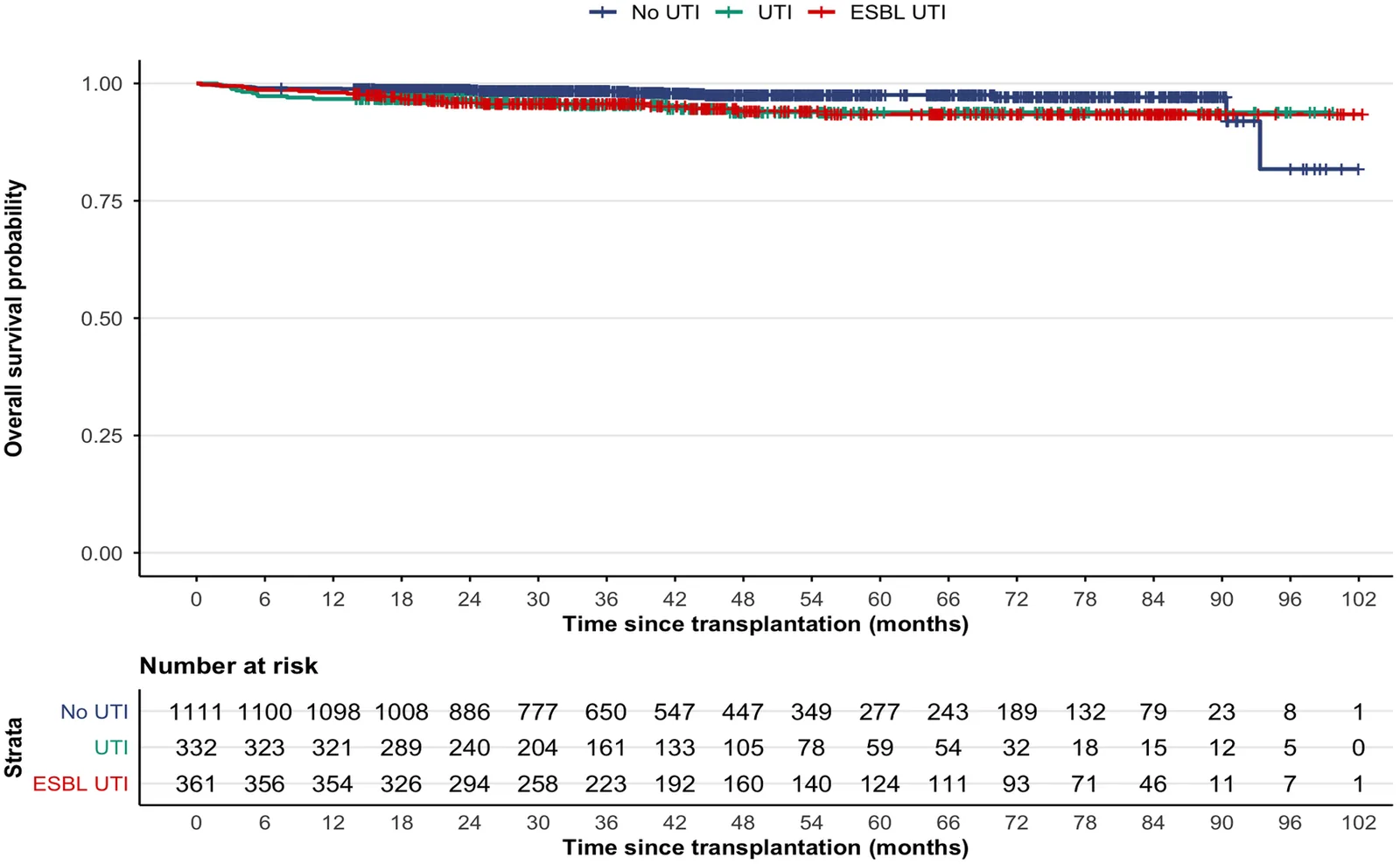
Long-term overall survival according to UTI status (extended follow-up to 102 months). This Kaplan–Meier curve illustrates overall survival following kidney transplantation, stratified by UTI status during the first year after transplantation (no UTI, non-ESBL UTI, and ESBL UTI), with extended follow-up up to 102 months.

**Figure 6:**
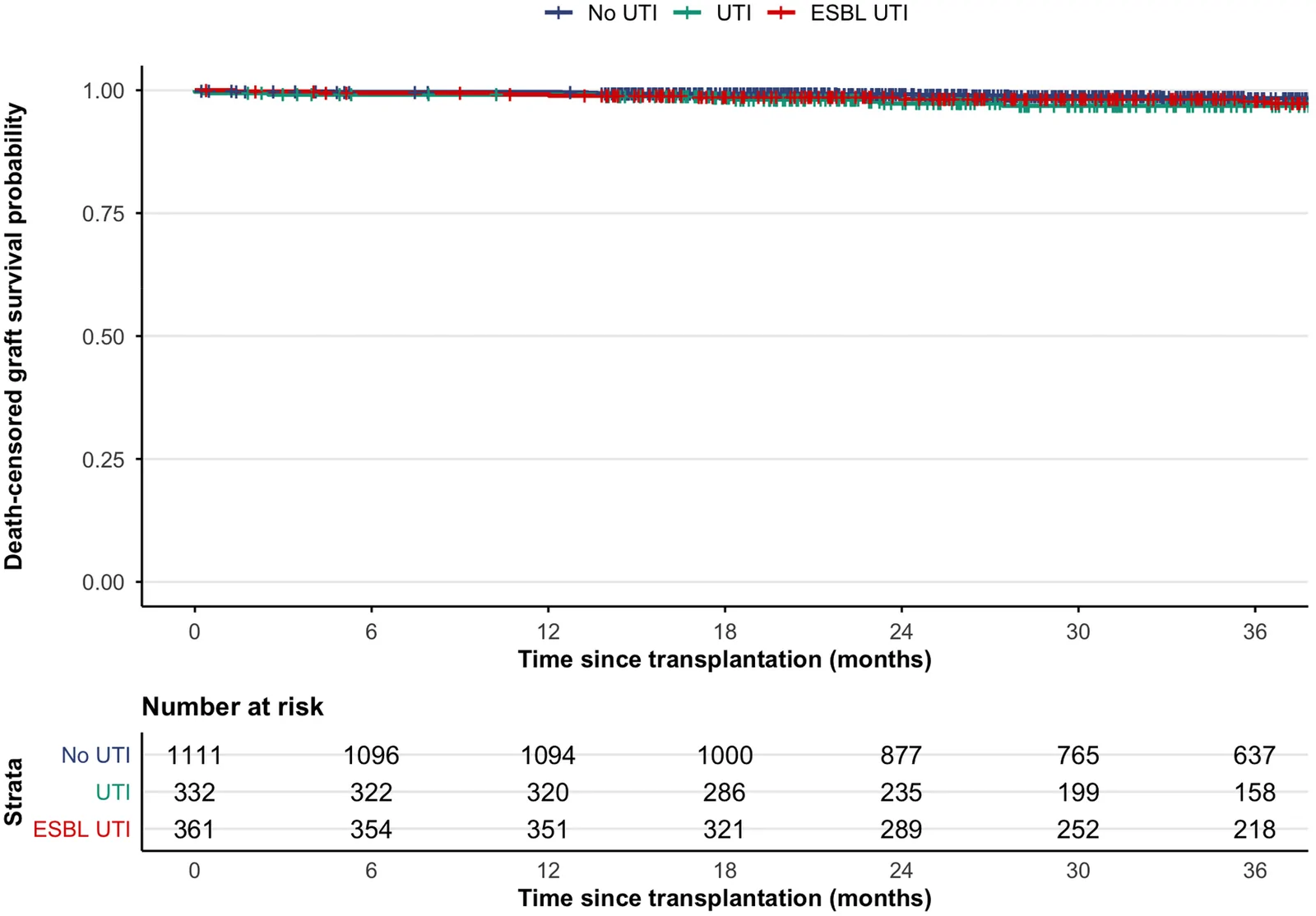
Three-year overall graft survival according to UTI status (with number at risk). This figure presents Kaplan–Meier estimates of overall survival up to 36 months following transplantation, stratified by UTI status during the first year after transplantation (no UTI, non-ESBL UTI, and ESBL UTI), along with the corresponding numbers at risk at predefined time points.

**Figure 7:**
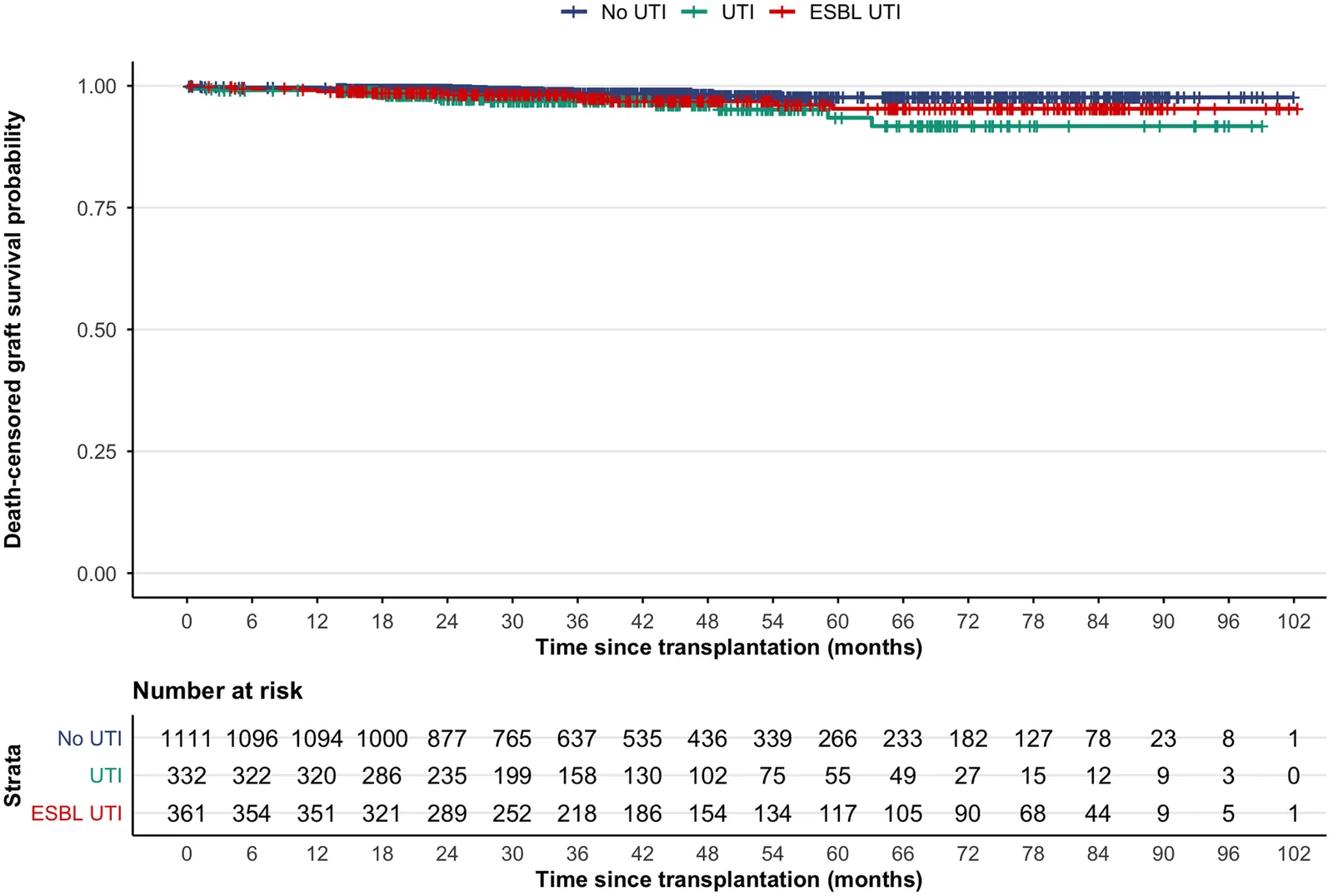
Long-term overall graft survival according to UTI status (extended follow-up to 102 months). This Kaplan–Meier curve illustrates overall survival following kidney transplantation, stratified by UTI status during the first year after transplantation (no UTI, non-ESBL UTI, and ESBL UTI), with extended follow-up up to 102 months.

The cumulative incidence of graft loss remained low over 3 years (<4%), with a numerically higher incidence in the ESBL group (3.1%) than in patients without UTI (1.2%), although this was not statistically significant (*p* = 0.20). Death-censored graft survival was highest in patients without UTI, while differences between ESBL and non-ESBL groups became more apparent over longer follow-up despite small absolute differences (Figures 3–7, Table 5).

**Table 5:** Time-aware associations of UTI and ESBL status with death-censored graft loss and all-cause mortality.

Analysis/exposure	Death-censored graft loss			All-cause mortality		
Model	aHR	95% CI	*p*	aHR	95% CI	*p*
Time-updated: any UTI	1.87	0.92–3.82	0.083	2.66	1.52–4.65	0.001
Time-updated first episode: non-ESBL UTI	1.88	0.84–4.19	0.125	2.34	1.21–4.52	0.011
Time-updated first episode: ESBL UTI	1.94	0.83–4.55	0.127	3.15	1.66–6.00	<.001
Day-365 landmark: non-ESBL UTI	1.62	0.64–4.09	0.311	2.79	1.24–6.28	0.013
Day-365 landmark: any ESBL UTI	2.51	1.02–6.20	0.046	1.40	0.48–4.13	0.542

aHRs: adjusted for age (per 10 years), sex, donor type, and pre-transplant UTI. Mortality time-updated models were stratified by donor type because donor type violated proportional hazards. Time-updated subtype analyses use first-episode ESBL status; the day-365 landmark uses any ESBL during year 1 among recipients under follow-up at day 365.

In time-updated models, any UTI was not clearly associated with death-censored graft loss (aHR 1.87, 95% CI 0.92–3.82; *p* = 0.083) but was associated with mortality in the donor-stratified model (aHR 2.66, 95% CI 1.52–4.65; *p* = 0.001). In the first-episode subtype sensitivity analysis, neither non-ESBL UTI (aHR 1.88, 95% CI 0.84–4.19; *p* = 0.125) nor ESBL UTI (aHR 1.94, 95% CI 0.83–4.55; *p* = 0.127) was clearly associated with graft loss. Both non-ESBL (aHR 2.34, 95% CI 1.21–4.52; *p* = 0.011) and ESBL first episodes (aHR 3.15, 95% CI 1.66–6.00; *p* < 0.001) were associated with mortality in the donor-stratified model. At the day-365 landmark, any first-year ESBL UTI was associated with later graft loss (aHR 2.51, 95% CI 1.02–6.20; *p* = 0.046) but not mortality (aHR 1.40, 95% CI 0.48–4.13; *p* = 0.542).

The time-updated any-UTI graft model included 40 events and five coefficients (8.0 events per coefficient); the donor-stratified mortality model included 60 events and four coefficients (15.0 events per coefficient). Global proportional-hazards tests were non-significant for these final models (*p* = 0.852 and *p* = 0.157, respectively), and all variance-inflation factors were ≤1.05. The day-365 landmark models remained event-limited (approximately five events per coefficient), so their estimates are exploratory.

## Discussion

In this large single-center cohort of 1,804 kidney transplant recipients, UTI within the first year after transplantation occurred in approximately 38% of patients. The majority of infections occurred early, with 57% developing within the first month post-transplant and 64% occurring while the ureteric stent was *in situ*. UTI was associated with an increased risk of acute rejection, graft loss, and mortality. ESBL infections were identified in approximately 48% of patients and were associated with a higher risk of recurrent episodes, as well as adverse outcomes including acute rejection and graft loss.

The incidence observed in our study is consistent with that reported in a large single-center study by Andrade-Sierra et al., who documented a UTI incidence of 42.5% (18), as well as with findings from two recent meta-analyses (19, 20). In contrast, our observed incidence is higher than that reported by Takai et al. (21), who found a UTI incidence of 26%, and Velioglu et al. (1), who reported a first-year incidence of 20.5%. This variability likely reflects differences in study populations, definitions of UTI, duration of follow-up, and local clinical practices.

We identified several risk factors for UTI, including female sex, older age, deceased donor transplantation, pre-transplant UTI, and post-transplant urological complications. Female sex has consistently been associated with an increased risk of UTI in transplant recipients (19, 20, 22–25). Similarly, older age (19, 20, 22–25) and deceased donor transplantation (19, 20, 25) have been linked to a higher incidence of post-transplant infections. The former likely reflects immunosenescence and a higher burden of comorbidities, whereas the latter may relate to delayed graft function, prolonged catheterization, and increased urological instrumentation. A history of UTI prior to transplantation is also a well-established risk factor for UTI (20, 25). Post-transplant urological complications, particularly ureteric stricture and leak, were among the strongest risk factors associated with UTI in our cohort, consistent with previous reports (1, 20, 23, 24). Collectively, these findings suggest that both patient- and transplant-related factors contribute to UTI risk, highlighting the importance of early risk stratification and targeted prevention in high-risk recipients.

In the present study, UTI was associated with higher rates of rejection (17.3% vs. 12.4%, *p* = 0.005), graft loss (3.5% vs. 1.4%, *p* = 0.005), and mortality (5.1% vs. 2.3%, *p* = 0.001), consistent with prior reports (24, 26, 27). However, existing data remain heterogeneous, largely due to small sample sizes, variable definitions, and limited adjustment for confounders. In our cohort, despite detailed phenotyping and multivariable adjustment, these findings require cautious interpretation. Recipients with UTI represented a higher-risk population characterized by older age, female sex, deceased donor transplantation, greater immunological burden, and more frequent urological complications—factors independently associated with adverse outcomes. While multivariable models were employed, residual confounding cannot be excluded, particularly given that urological complications both predispose to UTI and independently predict graft loss.

Our findings further suggest that the clinical impact is driven more by infection burden and recurrence than by isolated episodes, aligning with observations by Brune et al., who linked recurrent UTI to reduced long-term graft survival despite no difference in short-term outcomes (HR 4.41) (28) and Maanaoui et al., who associated graft pyelonephritis with graft loss but not mortality or rejection (29).

Although 1-year serum creatinine differed between groups, the absolute difference was small and of uncertain clinical significance. Therefore, UTI may function both as a potential contributor to adverse outcomes and as a marker of underlying recipient vulnerability and transplant complexity.

Nearly half (47.9%) of UTI-positive recipients developed ESBL-producing organisms within the first year, with 41.4% identified at the first episode. This rate is substantially higher than that reported by Ramadas et al. and Brakemeier et al. (8, 12), but aligns with findings of Espinar et al. (10) This discrepancy likely reflects regional antimicrobial resistance patterns and differences in study populations, and highlights the value of our large contemporary cohort in capturing the true burden of ESBL infections.

Gram-negative organisms predominated in our cohort, particularly Escherichia coli and Klebsiella species, with additional involvement of Enterococcus and Pseudomonas, consistent with prior studies and IDSA guidance (1, 9, 10, 30–32). In our cohort, baseline demographic and transplant-related characteristics did not differ significantly between ESBL and non-ESBL UTI groups, aligning with findings by Brune et al. (7) and Gołębiewska et al. (30) suggesting that pathogen-related factors may play a greater role in ESBL infection risk. In contrast, other studies have identified host and clinical factors-including older age, higher immunosuppression, acute rejection (8), diabetes, prior antibiotic exposure, previous UTI, and delayed graft function (10) -as important contributors. These discrepancies likely reflect differences in study populations and antimicrobial resistance patterns but collectively suggest that ESBL UTIs arise from a complex interplay of host vulnerability and microbial factors, rather than a single dominant predictor.

ESBL infections were associated with higher bacteremia rates (10.2% vs. 4.5%), supporting a more invasive phenotype consistent with prior studies linking ESBL bloodstream infections to urinary sources (11, 12, 33). Recurrent UTIs were common, with ESBL cases showing greater recurrence burden, often due to relapse (10), and higher recurrence rates reported in some cohorts (7, 9).

ESBL UTIs were associated with increased rejection and graft loss, consistent with some reports (26), while others found no significant differences (9). In our cohort, ESBL UTI was associated with death-censored graft loss after multivariable adjustment, although the absolute number of events was low and effect estimates were modest*.* Similarly, both ESBL and non-ESBL UTIs were associated with increased mortality, aligning with prior studies (6, 8, 10, 34) These findings should be interpreted cautiously given the low absolute event rates and the possibility of residual confounding from unmeasured factors—including prior antibiotic exposure, healthcare contact, colonization burden, and infection recurrence—, reverse causation, and time-dependent bias cannot be excluded in this retrospective analysis.

In contrast, Brune et al. (7), Bodro et al. (9) and Ramadas et al. (12) report no significant impact of ESBL UTI on graft loss, 1-year graft function or mortality, and Brakemeier et al. report superior graft survival in ESBL UTI (8). Overall, ESBL UTIs represent a more invasive and recurrent phenotype with potential adverse outcomes, although variability across studies suggests an influence of population and management factors.

We observed a low cumulative incidence of graft loss (<4% at 30 months), with slightly higher rates in recipients with ESBL UTI but no statistically significant difference between infection groups, while differences in mortality were more pronounced. These findings align with Brune et al_._ (7), who reported no significant impact of ESBL UTI on long-term graft or patient survival, and Ramadas et al. (12), who observed generally favorable graft outcomes despite ESBL infection.

Although the crude incidence of first UTI was substantially higher while the ureteric stent remained *in situ*, this difference disappeared after adjustment for time since transplantation. These findings may suggest that the apparent excess risk during the stented period is largely attributable to the early post-transplant period rather than ureteric stent itself. This period represents a time of increased infection susceptibility because of intensive immunosuppression, catheterization, and frequent urological interventions.

As one of the largest cohorts examining ESBL UTI in transplant recipients, our study demonstrates a markedly higher ESBL prevalence than the 10%–18% reported in Western cohorts and the global estimate of 10% (95% CI 4%–17%), highlighting a significant epidemiological shift. Importantly, both ESBL and non-ESBL UTIs independently associated with mortality, underscoring the broader clinical impact of UTI beyond antimicrobial resistance.

These findings support enhanced surveillance, region-specific antimicrobial stewardship, and targeted preventive strategies, particularly in high-prevalence settings, and emphasize the importance of addressing modifiable risk factors—including pre-transplant UTI and urological complications—to improve long-term transplant outcomes.

This study has several important strengths, including its large sample size, well-defined cohort, and use of strict diagnostic criteria. The extended observation period and comprehensive microbiological characterization allowed for detailed assessment of infection patterns.

However, this study has several important limitations. The retrospective design precluded collection of detailed antibiotic data, susceptibility profiles, and treatment information that may influence outcomes.

The single-center setting may limit generalizability to populations with different resistance patterns and clinical practices. Symptomatic UTIs could not be reliably distinguished from asymptomatic bacteriuria as data on total urine cultures performed and asymptomatic episodes were unavailable (especially beyond first 6 months), this potentially may affect the reported incidence and outcomes.

The observed associations between ESBL-producing organisms, recurrent infection, and adverse graft outcomes may reflect healthcare exposure and infection complexity rather than being an independent causal factor. This warrant further investigation, though antimicrobial susceptibility patterns and treatment-related factors could not be evaluated.

Given the observational design, the findings represent associations rather than causality, and residual confounding cannot be excluded.

## Conclusion

In this large kidney transplant cohort, UTI including ESBL-producing infections was common and associated with adverse outcomes, with a strong temporal relationship to ureteric stent indwelling. However, the observational design, low absolute event rates, and potential for time-dependent bias and residual confounding preclude causal inference. These findings underscore the early post-transplant period as a critical window for infection prevention and highlight the need for targeted strategies including early detection of resistant organisms, region-specific antimicrobial stewardship, and optimized peri-transplant urological management. Prospective studies are needed to clarify the independent contribution of antimicrobial resistance and ureteric stent practices to long-term transplant outcomes.

## Data Availability

The raw data supporting the conclusions of this article will be made available by the authors, without undue reservation.
